# A New Rapid and Sensitive Stability-Indicating UPLC Assay Method for Tolterodine Tartrate: Application in Pharmaceuticals, Human Plasma and Urine Samples

**DOI:** 10.3797/scipharm.1110-14

**Published:** 2011-12-05

**Authors:** Ramesh Yanamandra, Chandra Sekhar Vadla, Umamaheshwar Puppala, Balaram Patro, Yellajyosula. L. N. Murthy, Parimi Atchuta Ramaiah

**Affiliations:** 1 Analytical Development Laboratory, GVK Biosciences Private Limited, No.28A, Street No.15, IDA, Nacharam, Hyderabad-500 076, India; 2 Department of Organic Chemistry, Food, Drugs & Water, Andhra University, Visakhapatnam-530 003, India

**Keywords:** Column liquid chromatography, Ultra Performance Liquid Chromatography, UPLC, Tolterodine Tartrate, Validation and quantification

## Abstract

A new rapid, simple, sensitive, selective and accurate reversed-phase stability-indicating Ultra Performance Liquid Chromatography (RP-UPLC) technique was developed for the assay of Tolterodine Tartrate in pharmaceutical dosage form, human plasma and urine samples. The developed UPLC method is superior in technology to conventional HPLC with respect to speed, solvent consumption, resolution and cost of analysis. Chromatographic run time was 6 min in reversed-phase mode and ultraviolet detection was carried out at 220 nm for quantification. Efficient separation was achieved for all the degradants of Tolterodine Tartrate on BEH C18 sub-2-μm Acquity UPLC column using Trifluoroacetic acid and acetonitrile as organic solvent in a linear gradient program. The active pharmaceutical ingredient was extracted from tablet dosage form using a mixture of acetonitrile and water as diluent. The calibration graphs were linear and the method showed excellent recoveries for bulk and tablet dosage form. The test solution was found to be stable for 40 days when stored in the refrigerator between 2 and 8 °C. The developed UPLC method was validated and meets the requirements delineated by the International Conference on Harmonization (ICH) guidelines with respect to linearity, accuracy, precision, specificity and robustness. The intra-day and inter-day variation was found be less than 1%. The method was reproducible and selective for the estimation of Tolterodine Tartrate. Because the method could effectively separate the drug from its degradation products, it can be employed as a stability-indicating one.

## Introduction

Ultra Performance Liquid Chromatography (UPLC) system is an innovative technique that brought revolution in high performance liquid chromatography by outperforming conventional HPLC. UPLC decreases sample run times up to a factor of 10, uses up to 95% less solvent and significantly improves productivity in the lab as compared to HPLC. The sub-2-μm hybrid particle chemistry, which offers significant benefits over today’s HPLC systems, equipped with standard 5-μm particle chemistries. UPLC achieves the speed by using novel sub two-micron particles that reduce chromatographic run times and improve resolution.

UPLC was designed as a total system to leverage both ultra-high pressure and small particle separation attributes that result in uniquely superior performance with significant improvements in resolution, sensitivity and speed. UPLC system allows chromatographers to work at higher efficiencies with a much wider range of linear velocities, flow rates and backpressures. UPLC Photodiode Array (PDA) Detector detects and quantifies lower concentrations of sample analyte and trace impurities with maximum sensitivity. The present study was conducted to quantify Tolterodine Tartrate in pharmaceutical dosage form, human plasma and in urine samples by using RP-UPLC technique.

Tolterodine Tartrate (TTT) (CAS NO: 124937-52-6), 2-[3-(di-*iso*-propylamino)-1-phenyl-propyl]-4-methylphenol, is listed in the Merck Index [1] and exists in two isomeric forms (R) and (S). It is not official in IP, BP and USP. Tolterodine Tartrate is a potent muscarinic receptor antagonist used in the treatment of urinary urge incontinence and other symptoms of an overactive bladder [2]. Various spectrophotometric methods [3, 4] and a few HPLC analytical methods for the stability-indicating and quantification of Tolterodine [5–7] and in plasma [8–11] for dosage form have been reported. An enantio-specific HPLC method for the determination of (S)-enantiomer impurities in (R)-Tolterodine Tartrate [12] and a validated chiral HPLC method for the separation of enantiomers [13], HPLC analytical method for the determination of related substances [14–16] have been reported. TTT is available in the commercial market in tablet and capsule dosage form by different manufacturers. Present study involves a new rapid, sensitive and validated UPLC method for the assay of TTT for tablet dosage form and its application in spiked human plasma and urine samples.

A review of the literature did not reveal the presence of assay of TTT dosage form by using UPLC technique. The reported HPLC methods are more time consuming, complex mobile phase mixtures, use high flow rate of analysis, lack of sensitivity and peak symmetry, and not compatible to LC-MS technique. The aim of this work was to develop a suitable UPLC method for the assay of TTT by using UV detection which is fast, uses less flow rate, less time consuming with better sensitivity, peak symmetry and is compatible with LC-MS technique thus able to provide additional structural information. The developed assay method can quantify TTT in tablet dosage form in the presence of its degradation products, in spiked human plasma and urine samples.

## Experimental

### Chemicals

Bulk sample of Tolterodine Tartrate was obtained from Inogent Laboratories Ltd., (Hyderabad, India). Commercially available Roliten manufacture by Ranbaxy Laboratories Ltd. was purchased from a local pharmacy containing Tolterodine Tartrate (2 mg). HPLC grade acetonitrile was obtained from Apchem, Ashonuj Chem Pvt. Ltd (Navi Mumbai, India). Trifluoroacetic acid (TFA) spectroscopy grade was purchased from Merck (Darmstadt, Germany). High purity water was obtained from Millipore Milli-Q Plus water purification system. Acquity BEH-C18 and BEH-Shield RP18 columns were purchased from Waters.

### Equipment

The LC system used for method development and method validation consisted of a Waters Acquity UPLC with PDA Detector with a separation module. Empower software (Waters) was used for data handling installed on a Pentium computer (Lenovo).

### Chromatographic Conditions

The analysis was carried out on Acquity BEH C18 column 100 mm × 2.1 mm, 1.7μm. The mobile phase composition was 0.025% TFA (aqueous) buffer as mobile phase A and 0.025% TFA in Acetonitrile as mobile phase B which was filtered through 0.45 μm membrane filter and sonicated for 15 min. A linear time gradient program [Time/%B: 0/30, 4/80, 6/80, 6.1/30] at a flow rate of 0.30 mL min^−1^, detection at 220 nm, and chromatographic run time of 6.0 min was used. The injection volume was 3.0 μL, a mixture of Acetonitrile and Water (50:50, v/v) was used as diluent. Prior to injection of the drug solution, the column was equilibrated for at least 15 min with the initial time gradient mobile phase conditions flowing through the system.

### Preparation of Solutions

#### Preparation of Standard Solution

Bulk Standard of TTT was prepared by dissolving 20 mg in 100 mL standard volumetric flask containing approximately 50 mL of diluent and the solution was sonicated for 10 min, and the volume was made up to the mark with diluent to obtain a concentration of 200 μg mL^−1^. Subsequent dilution of this solution was made with diluent to obtain the concentration of 40 μg mL^−1^.

#### Preparation of Sample Solution

Ten tablets were weighed to determine the average tablet weight and powdered in a mortar. Powder equivalent to 20 mg of TTT was transferred into a 100 mL volumetric flask. About 50 mL of diluent was added and kept on a rotary shaker for 20 min to disperse the material completely, followed by sonication for 10 min, cooled to room temperature, made up to mark with diluent and mixed well. About 25 mL of sample solution was centrifuged for 15 min at 2,500 rpm. The sample solution was filtered through a 0.45 μm Nylon-66 membrane syringe filter, and 10.0 mL of this solution was taken in a 50 mL volumetric flask and made up to volume with diluent.

#### Plasma drug sample preparation and extraction, and urine sample preparation

Blood samples were collected from healthy volunteers and then centrifuged at 5000 rpm for 10 min. The supernatant obtained was stored at −20 °C. After thawing, plasma samples are spiked with working solutions to produce desired concentrations of the drug. To 0.5 mL of TTT spiked plasma solution added 2.5 mL of extraction solvent [Ethyl Acetate:n-Hexane, 70:30 v/v], cyclomixed for 10 min and centrifuged the sample solution for 10 min at 2500 rpm. 2 mL of supernatant layer was taken and evaporated under Nitrogen at 50 °C for 20 min and reconstitute the sample with 0.5 mL of diluent. A 3.0 μL volume of each sample was injected and chromatographed under the above conditions.

### Method Validation

Method validation was performed according to International Conference on Harmonization (ICH) guidelines with respect to precision, linearity, accuracy, specificity and robustness [17].

#### Precision

The precision of the developed method was evaluated by six replicate injections of the above standard mixture. The RSD (Relative Standard Deviation) was evaluated for peak areas, USP (United States of Pharmacopeia) tailing factor and plate count for TTT. The intermediate precision of the method was also evaluated on a different column.

#### Linearity and range

Stock solution TTT 200 μg mL^−1^ was prepared by dissolving the appropriate amount in 100 mL of diluent and further diluted to the required concentrations with diluent. The solution was prepared at six concentration levels ranging from 25% to 150% of the target concentration 40 μg mL^−1^. Linearity of the method was studied by injecting six concentrations of the drug prepared in the diluent in triplicate into the UPLC system keeping the injection volume constant. The peak areas were plotted against the corresponding concentrations to obtain the calibration graphs. The correlation coefficients, slopes and *Y*-intercepts of the calibration curve were determined.

#### Accuracy

Standard addition and recovery experiments were conducted to determine accuracy of the method for the quantification of TTT. The study was carried out at 50%, 100% and 150% for three replicate injections of each concentration of the analyte followed by calculation of the percentage recovery.

#### Solution Stability and Mobile Phase Stability

Solution stability of TTT in a tightly capped volumetric flask for 40 days when stored in a refrigerator between 2 to 8 °C temperature was studied and the content of the drug was determined. Mobile phase stability was assessed over a period of 72 h by injecting the freshly prepared sample solutions in 8 h interval. The content of the drug was determined in the test solution.

#### Specificity

The specificity of the analytical method was checked in different conditions of acid hydrolysis (0.1M HCl), base hydrolysis (0.1M NaOH) and peroxide treatment (0.3% H2O2). Aliquot quantity of TTT stock solution was taken in different volumetric flasks and added 3.0 mL of 0.1M HCl (0.018%), 3.0 mL of 0.1M NaOH (0.024%), and 1.0 mL of 0.3% H2O2 (0.006%) respectively and diluted to 50 mL with diluent. These solutions were refluxed at 80 °C for 3 h, 6 h and 8 h, cooled to room temperature, made up to 100 mL with diluent and analyzed by UPLC.

#### Robustness & Ruggedness

The experimental conditions were deliberately altered and the impact on retention time, peak asymmetry and % assay was evaluated by changing flow rate, buffer concentration, column temperature, buffer pH and mobile phase composition. The effect of different column, different analyst, and different system was also studied as part of the ruggedness of the method.

#### Plasma and Urine drug analysis

The availability of TTT in tablet dosage form in human plasma and urine was determined by the stated chromatographic conditions. Blood samples of healthy volunteers (age group between 23–26 years, non-smokers, and not taking any other medicines) were collected. Multiple blood samples were collected in evacuated glass tubes through an internal cannula placed in the forearm veins or directly from a vein. The blood was then slightly shaken and centrifuged at 5000 rpm for 10 min and the plasma was separated. The obtained plasma samples were stored at −20 °C. The plasma samples were thawed at room temperature, spiked with TTT, extracted back, reconstituted and assayed the concentration levels. The urine samples of healthy volunteers were collected and spiked with TTT tablet dosage form of desired concentration followed by dilution with the diluent. The assay results for plasma and urine samples are summarized in Table 5 & 6.

## Results and Discussion

### UPLC Method Development and Optimization

Development of a rapid, rugged and suitable UPLC method for the quantification of TTT required a number of trials to be carried out using different mobile phase compositions. As part of the preliminary work, separation was attempted on Acquity BEH C18 (100 mm × 2.1 mm, 1.7 μm) column with 0.05M Ammonium Acetate in aqueous as buffer and acetonitrile as organic in different gradient programs at a flow rate of 0.3 mL min^−1^. Under these conditions the peaks found were because of Tartaric acid and Tolterodine free base. This was confirmed by separately injecting Tartaric acid and Tolterodine. Attempts were also made with 0.05% Formic acid (acidic) and 0.01M Ammonium bicarbonate (basic) with pH ranges 3 to 7 were studied to control the dissociation of TTT. To minimize the dissociation of TTT, and to improve the peak shape, attempts were made with different percentages of TFA buffer and found 0.025% TFA (pH-2.3) as a suitable buffer. Trials were also done at different flow rates and different temperatures to optimize the peak shape, sensitivity, tailing factor and plate count. 0.025% TFA in aqueous and 0.025% TFA in acetonitrile worked out best combination for this method. A mixture of Acetonitrile and Water (50:50 v/v) was used as diluent and detection was monitored at 220 nm.

The typical retention time of TTT is 2.4 min in a total chromatographic run time of 6 min. The resolution between TTT and the degradants generated after stress degradation were found to be good in the developed RP-UPLC assay method. The system suitability test results are summarized in Table 1.

Representative assay chromatograms of Tolterodine Tartrate (TTT):

Potential interferences from excipients were investigated and no interferences from excipients observed in the chromatography. Analyses were performed for three different batches of tablet dosage form (each *n* = 3). In all batches the contents of TTT were within the limits of ≥ 90.0% and ≤ 110.0% (w/w). The assay result for tablet dosage form, plasma, and urine samples is summarized in Table 4, 5, & 6.

### Method Validation

#### Precision

The repeatability was checked by repeatedly injecting (n = 6) solution of TTT and the area RSD values for TTT was found to be within 2.0% confirming a suitable precision of the method. System suitability and system precision results are summarized in Table 1. Intra-day and inter-day precision were evaluated by analyzing six replicate injections of assay concentration and assay results are summarized in Table 3.

#### Linearity

The linear regression equation is y = 6622 × − 8088 and the correlation coefficient obtained was greater than 0.99 for tablet dosage form which confirmed the linear relationship between peak areas and concentrations. Slope and *Y*-Intercept values were 6622 and 8088. Linearity was determined for six concentrations of each three replicate injections. The linearity test results and linearity curve is summarized in Table 1 & 2.

#### Accuracy

The percentage recovery obtained ranged from 90.0% and 110.0% for tablet dosage form. The accuracy test results are summarized in Table 1.

#### Solution Stability and Mobile phase Stability

The stability of TTT was assessed during analysis and storage. No significant changes were observed in the content of TTT in mobile phase stability experiments. The standard, sample solutions prepared in clear volumetric flasks were stable up to 40 days in temperature from 2 to 8 °C. All urine sample solutions were stored in a refrigerator between 2 and 8 °C and all plasma sample solutions were stored at −20 °C.

#### Specificity

Degradation was not found in acid hydrolysis (at 80°C, up to 8 hours) which confirms that TTT was found to be stable to acidic hydrolysis. The drug was found to be liable to base hydrolysis as a total of 73% degradation was found (at 80°C, up to 8 h) with a maximum individual degradation product of 72%. Degradation was found in peroxide treatment of 38.5%, 4%, 14%, 1.28% and 2.06% at relative retention times of 0.32, 0.35, 1.04, 1.62 and 1.75 min respectively. As more degradation products were found in base hydrolysis and peroxide hydrolysis, the hydrolysis conditions were reduced to 3 h by maintaining the temperature at 80°C. Under these conditions no degradation was found in peroxide hydrolysis, whereas 33% degradation was found in base hydrolysis at 0.30 relative retention time. The tablet dosage form showed no degradation products under dark, light, heat and also in freeze-thaw cycles. Peroxide hydrolysis conditions were established at 80°C for 6 h, under these conditions 34% total degradation was found out of which 9.11%, 1.54%, 20.56%, 0.82%, and 1.22% degradation at relative retention of 0.30, 0.34, 1.03, 1.62, 1.75 min, respectively. The method was specific because degradants were separated to base line from main component and the peak purity flag passes for tablet dosage form which confirms the method is selective and homogeneity of the drug product.

#### Detection and quantification limit

The analytical sensitivity of the method was anticipated from the signal to noise ratio 3:1 for LOD and 10:1 for LOQ. The minimum limits at which the analytes could be readily detected and quantified were summarized in Table 1.

#### Robustness

Deliberate changes in chromatographic conditions (different flow rate, buffer concentration, column temperature, buffer pH, organic solvent %) have no impact on the assay of the drug product which shows good robustness of the method. The robustness test results are summarized in Table 1.

## Conclusion

In this study, an accurate, rapid, simple, sensitive, reproducible and stability-indicating reversed-phase UPLC method with UV detection was described for the assay of Tolterodine Tartrate. The method was fully validated and applied successfully to quantify the drug in pharmaceutical dosage form, human plasma and in urine samples. A short chromatographic run time of 6 min allows the quantification of Tolterodine Tartrate in bulk raw material, tablet dosage form in quality control laboratories, its application in biological samples, and is compatible with LC-MS technique where there is no need for traditional HPLC methods with complex mobile phase mixtures, long chromatographic run times and more solvent consumed methods. The developed RP-UPLC technique will eliminate significant time and cost per sample from analytical process while improving the quality of results. The proposed method is not hazardous to human health or to the environment and is more economic because a large number of samples can be analyzed in a short period of time.

## Figures and Tables

**Fig. 1:**
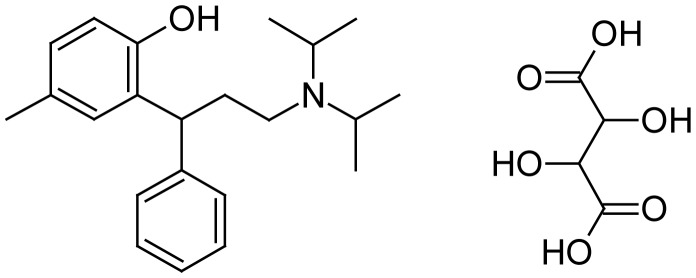
Structure of Tolterodine Tartrate

**Fig. 2:**
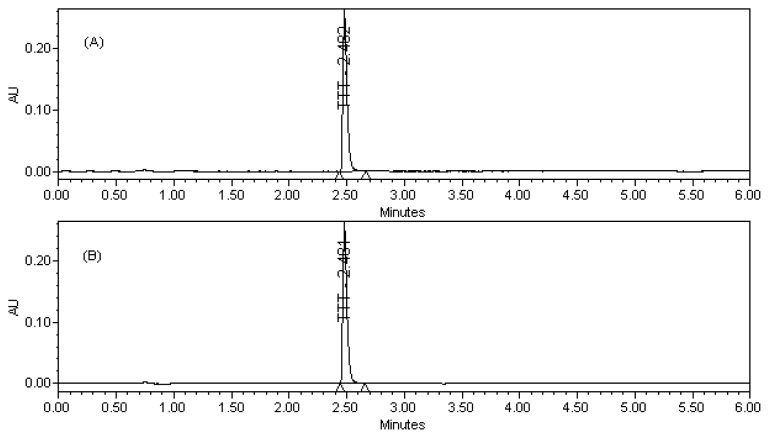
(A) TTT bulk raw material (B) TTT tablet dosage form

**Fig. 3:**
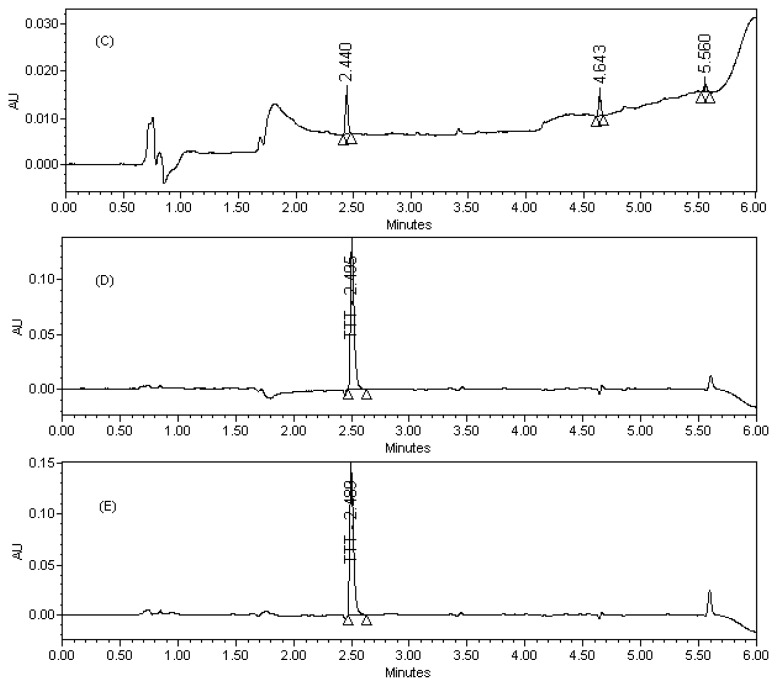
(C) Blank plasma (D) TTT bulk raw material spiked plasma (E) TTT tablet spiked plasma

**Fig. 4:**
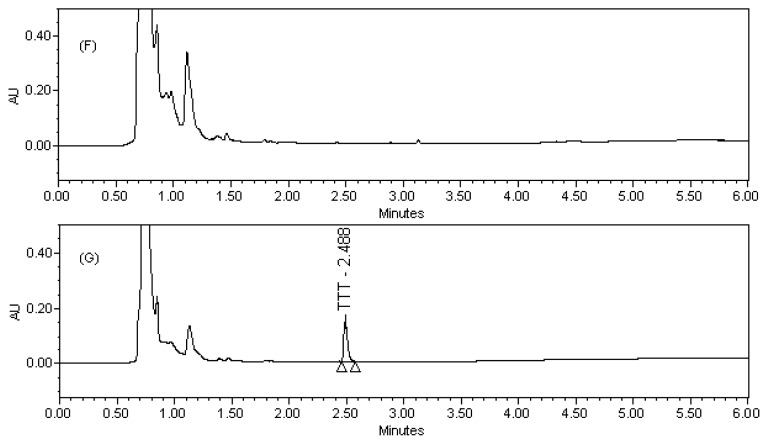
(F) Blank urine sample (G) TTT tablet spiked urine sample

**Fig. 5:**
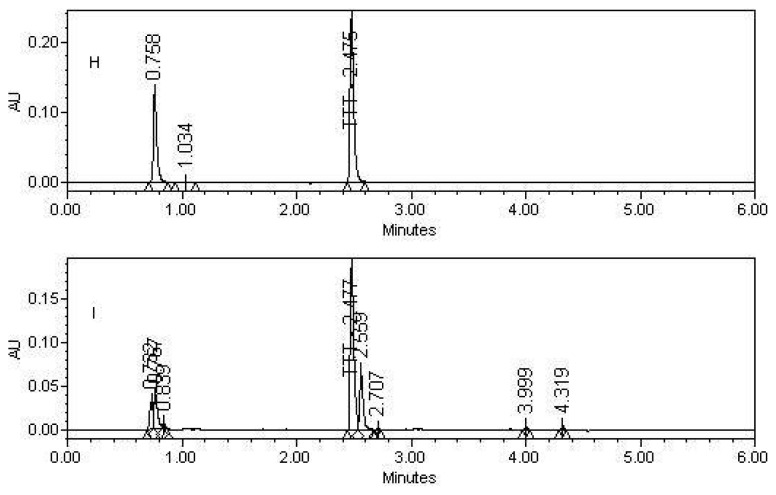
(H) Sodium hydroxide hydrolysis (I) Hydrogen Peroxide hydrolysis

**Tab. 1:** System suitability, Precision, Linearity, Accuracy, LOD, LOQ and Robustness of the proposed method

System suitability & Precision				
*Rt*		2.60	Capacity factor	1.60
*N*		*46728*	% RSD (n = 6)	0.63
*USP Tailing*		*1.14*		

**Linearity**				

Correlation coefficient (r)		0.999	Intercept	8088
Slope		6622.3	% RSD for Intercept	8.68
% RSD for slope		0.16		

**Accuracy (n = 3)**			**(% Recovery ± % RSD)**	

50%			99.9 ± 0.20	
100%			102.2 ± 0.05	
150%			103.6 ± 0.04	
Mean (n = 3)			101.9 ± 1.83	

**Limit of Detection (LOD) and Limit of Quantification (LOQ)**				

LOD		0.05 μg mL^−1^	LOQ	0.15 μg mL^−1^

**Robustness testing**^a^ **(n = 3)**				

**Factor**^a^	**Level**	**Retention time**	**Asymmetry**	**% Assay**

**Tolterodine Tartrate**				

A: Flow rate (mL min^−1^)				

0.25	−1	2.88	1.17	101.50
0.30	0	2.47	1.14	100.32
0.35	+1	2.33	1.05	101.41
Mean ± SD (n = 3)		2.56 ± 0.29	1.12 ± 0.06	101.07 ± 0.67

B: Buffer concentration				

0.020%	−1	2.41	1.10	98.95
0.025%	0	2.45	1.14	100.32
0.030%	+1	2.48	1.16	99.90
Mean ± SD (n = 3)		2.45 ± 0.04	1.13 ± 0.03	99.72 ± 0.68

C: Column Temperature				

23 °C	−1	2.50	1.15	98.00
25 °C	0	2.45	1.12	99.21
27 °C	+1	2.40	1.09	97.80
Mean ± SD (n = 3)		2.45 ± 0.05	1.12 ± 0.03	98.33 ± 0.76

D: Buffer pH				

2.2	−1	2.42	1.08	98.23
2.3	0	2.45	1.12	99.21
2.4	+1	2.50	1.15	98.28
Mean ± SD (n = 3)		2.46 ± 0.04	1.12 ± 0.04	98.57 ± 0.55

E: % of acetonitrile in the mobile phase (v/v)				

27	−1	2.81	1.17	98.30
30	0	2.45	1.12	99.21
33	+1	2.17	1.11	98.88
Mean ± SD (n = 3)		2.48 ±0.32	1.13 ±0.03	98.80 ± 0.46

a Three factors were slightly changed at three levels (−1, 0, 1); n…number of determinations; *R_t_…*retention time; *N*…number of theoretical plates; TTT… Tolterodine Tartrate.

**Tab. 2:** Linearity curve of Tolterodine Tartrate in RP-UPLC

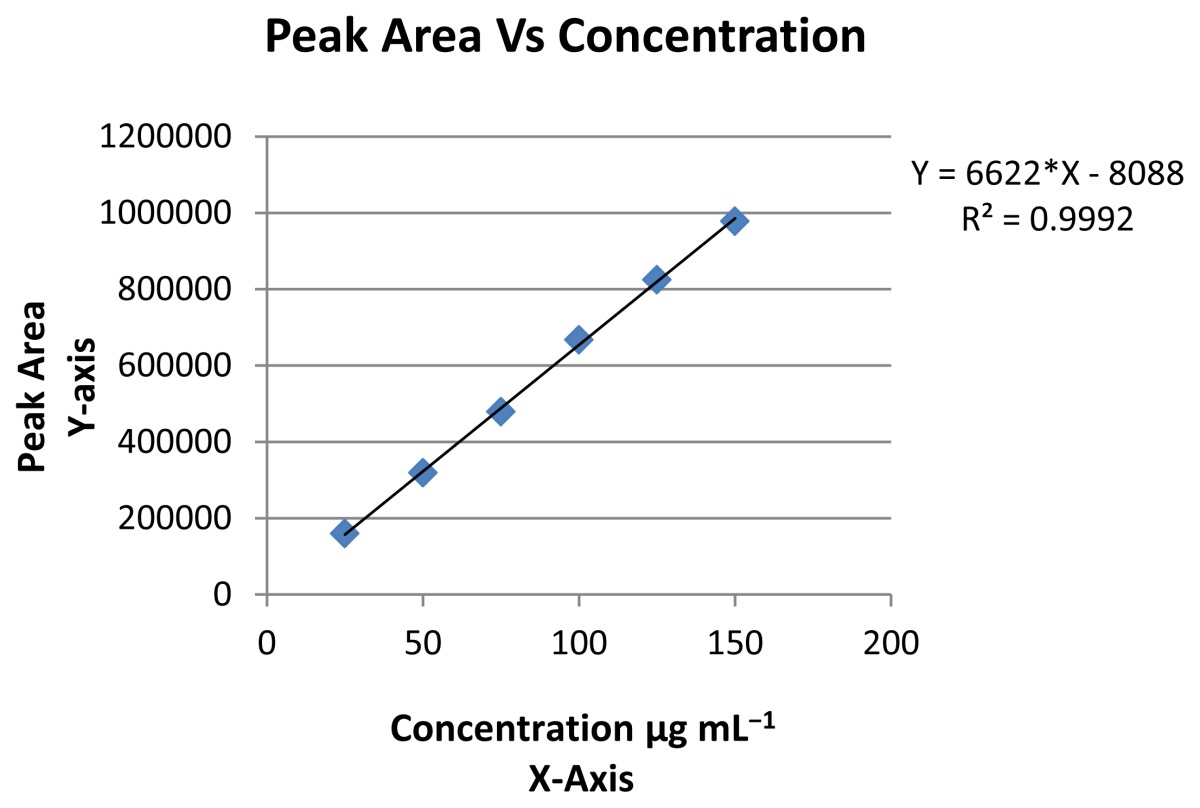

**Tab. 3:** Precision of the proposed UPLC method

Concentration (μg mL^−1^)	Concentration found	

	Intra-Day	Inter-Day
40	40.32 ± 0.35	40.17 ± 0.39
Mean (n = 3) ± % RSD		

**Tolterodine Tartrate (μg mL^−1^)**	**Intermediate precision (n=6)**	

	**Measured conc. ± SD**	**Recovery (%) ± (%) RSD**

20	20.088 ± 0.39	100.44 ± 0.39
40	40.276 ± 0.35	100.69 ± 0.35
60	60.384 ± 0.37	100.64 ± 0.36

**Tab. 4:** Assay results for tablet dosage form by the proposed method

Tolterodine Tartrate (2 mg)	Tolterodine Tartrate found (mg per tablet)	

	Mean ± SD (n=3)	Recovery (%)
Dosage form-B.No: 1	2.027 ± 0.22	101.38
Dosage form-B.No: 2	2.006 ± 0.06	100.34
Dosage form-B.No: 3	2.015 ± 0.16	100.76

**Tab. 5:** Assay results for plasma samples by the proposed method

Tolterodine Tartrate	Amount spiked (mg)	Amount found (mg)	

		Mean ± SD (n=3)	Recovery (%)
Bulk-1^st^ Lot	2	1.924 ± 0.22	96.23
Bulk-2^nd^ Lot	2	1.971 ± 0.51	98.53
Dosage form-B.No: 1	2	1.982 ± 0.31	99.10
Dosage form-B.No: 2	2	2.005 ± 0.12	100.26

n…determinations; SD…standard deviation.

**Tab. 6:** Assay results for urine samples by the proposed method

Tolterodine Tartrate	Amount spiked (mg)	Amount found (mg)	

		Mean ± SD (n=3)	Recovery (%)
Dosage form-B.No: 1	2	1.912 ± 0.26	95.60
Dosage form-B.No: 2	2	1.956 ± 0.51	97.79
